# Is systematic fecal carriage screening of extended-spectrum beta-lactamase-producing *Enterobacteriaceae* still useful in intensive care unit: a systematic review

**DOI:** 10.1186/s13054-019-2460-3

**Published:** 2019-05-14

**Authors:** Renaud Prevel, Alexandre Boyer, Fatima M’Zali, Agnès Lasheras, Jean-Ralph Zahar, Anne-Marie Rogues, Didier Gruson

**Affiliations:** 1 CHU Bordeaux, Medical Intensive Care Unit, Pellegrin Hospital, F-33000 Bordeaux, France; 2 UMR 5234 CNRS, Bordeaux University, F-33000 Bordeaux, France; 3 Univ. Bordeaux, CHU Bordeaux, Hygiène hospitalière, F-33000 Bordeaux, France; 4 Unité INSERM - IAME UMR 1137, Université Paris-13, Bobigny, France; 5 Univ. Bordeaux, Inserm, Bordeaux Population Health Research Center, team pharmacoepidemiology, UMR 1219, F-33000 Bordeaux, France; 6 Bordeaux, France

**Keywords:** Extended-spectrum beta-lactamase, Carriage, Screening, Intensive care, Cross-transmission, Nosocomial infections

## Abstract

**Background:**

Extended-spectrum beta-lactamase-producing *Enterobacteriaceae* (ESBL-E) are disseminating worldwide leading to increased hospital length of stay and mortality in intensive care units (ICU). ESBL-E dissemination was first due to outbreaks in hospital settings which led to the implementation of systematic fecal carriage screening to improve hygiene procedures by contact precautions. ESBLs have since spread in the community, and the relevance of contact precautions is questioned. ESBL-E dissemination led to an overuse of carbapenems triggering the emergence of carbapenem-resistant *Enterobacteriaceae*. Empirical antimicrobial therapy based on ESBL-E fecal carriage has been proposed but is debated as it could increase the consumption of carbapenems among ESBL-E carriers without any clinical benefit. Finally, selective decontamination among ESBL-E fecal carriers is evoked to decrease the risk for subsequent ESBL-E infection, but its efficacy remains debated. We propose to systematically review the evidence to recommend or not such systematic ESBL-E fecal carriage screening in adult ICU.

**Methods:**

Every article focusing on ESBL-E and ICU available on the MEDLINE database was assessed. Articles were included if focusing on cross-transmission, efficacy of hygiene procedures, link between ESBL-E colonization and infection or guidance of empirical therapy or selective decontamination efficacy.

**Results:**

Among 330 articles referenced on PubMed, 39 abstracts were selected for full-text assessment and 25 studies were included. Systematic screening of ESBL-E fecal carriage to guide contact precautions do not seem to decrease the rate of ESBL-E cross-transmission. It has a very good negative predictive value for subsequent ESBL-E infections but a positive predictive value between 40 and 50% and so does not help to spare carbapenems. Cessation of ESBL-E carriage systematic screening could decrease the use of carbapenems in ICU without any clinical harm. Nevertheless, further studies are needed to validate these results from monocentric before-after study. Selective decontamination strategy applied to ESBL-E fecal carriers could be helpful, but available data are conflicting.

**Conclusion:**

Current knowledge lacks of high-quality evidence to strongly recommend in favor of or against a systematic ESBL-E fecal carriage screening policy for ICU patients in a non-outbreak situation. Further evaluation of selective decontamination or fecal microbiota transplantation among ESBL-E fecal carriers is needed.

**Electronic supplementary material:**

The online version of this article (10.1186/s13054-019-2460-3) contains supplementary material, which is available to authorized users.

## Background

The increasing antimicrobial resistance remains a major threat worldwide [1]. Extended-spectrum beta-lactamase-producing *Enterobacteriaceae* (ESBL-E) fecal carriage is increasing, especially in long-term care facilities and ICU [2–4]. The ESBL-E dissemination is of paramount importance since ESBL-E infections lead to increased healthcare costs, length of stay and mortality [5–8]. ESBL-E dissemination was first due to clonal outbreaks in hospital settings of TEM- and SHV-producing *Enterobacteriaceae*, and cross-transmission was highly involved in the outbreaks in ICU leading to the enforcement of hygiene procedures [9–11]. Systematic ESBL-E fecal carriage screening in ICU has been proposed as a standard of care by some societies as the provided information was thought to be useful to guide hygiene procedures [9]. More recently, ESBL gene epidemiology has been totally overhauled by the emergence of CefoTaXimase-München (CTX-M) enzymes which became the most predominant ESBL type worldwide in the early 2000s [12]. Contact precautions were still recommended for hospitalized ESBL-E fecal carriers to prevent nosocomial spread [13]. Therefore, systematic ESBL-E fecal carriage screening at admission was still considered as a standard of care to reduce guide contact precautions and decrease the incidence of hospital-onset ESBL-E clinical isolates [14, 15]. Unfortunately, despite those precautions, a steady increase of ESBL-E rate has been reported in hospital settings but also in the community which can range from 1–6% in Europe and North America to 60% in India [2, 16]. The paradigm of ESBL-E dissemination occurring only in hospital settings by clonal outbreaks has been dramatically changed with the ESBL-E dissemination now occurring everywhere, both in community and hospital settings, enhancing the need for further evaluation of contact precautions’ efficacy.

Another interest of systematic screening of ESBL-E fecal carriage in ICU could also be to guide the empiric treatment of ICU-acquired infections. In fact, this treatment is often challenging, and carbapenems have emerged as the gold standard as they are almost always active on ESBL-E. However, carbapenems use leads to the emergence of *Enterobacteriaceae* resistant to carbapenems including non-fermenting Gram-negative, which cause almost half of culture-positive ventilator-associated pneumonia (VAP) in ESBL-E carriers [17–19]. As a consequence, alternative strategies to spare carbapenems are urgently needed [20]. ESBL-E fecal carriage has been suggested as a tool to guide the prescription of carbapenems for empiric antimicrobial therapy because of the suspected link between colonization and infection. Nevertheless, the predictive value of ESBL-E fecal carriage for helping the clinician to tailor the empirical antimicrobial therapy and its impact on the use of carbapenems is still a matter of debate.

Systematic fecal carriage screening of ESBL-E could also help to guide decolonization procedures. As a matter of fact, gut microbiota is now considered as the main source of ESBL-E dissemination [21]. Changes in the composition of the gut flora, due in particular to antibiotics and critical illness, can happen silently, leading to the selection of highly resistant bacteria including ESBL-E which can remain for months in the gut of the carrier without causing any symptoms or translocate through the gut epithelium, induce healthcare-associated infections, undergo cross-transmission to other individuals, and cause limited outbreaks. However, data about gut microbiota modulation (by selective decontamination or by fecal microbiota transplantation (FMT)) to eradicate ESBL-E fecal carriage are scarce.

We propose here to systematically review the evidence to recommend or not such systematic ESBL-E fecal carriage screening in ICU regarding the guidance of hygiene procedures, of empirical antimicrobial therapy for ICU-acquired infections and of selective decontamination strategy.

## Methods

### Search strategy

We searched the MEDLINE database for English language articles published from the inception of the database to February 15, 2019. A combination of MeSH/Emtree and title/abstract keywords was used. The search terms were “ESBL,” “ESBL-E,” “ESBLE,” “extended spectrum beta-lactamase,” “ICU” with non-relevant terms “neonatal,” “pediatric,” “children,” and “infants”.

### Eligibility criteria

Studies were considered suitable for inclusion in this systematic review if (1) they enrolled ICU ESBL-E fecal carriers in a non-outbreak situation, (2) they assess the rate of ESBL-E cross-transmission in ICU, (3) they evaluate the efficacy of contact precautions to limit the spread of ESBL-E, (4) they assess the link or the prognostic value of ESBL-E carriage for subsequent ESBL-E infection, (5) they assess the efficacy of selective decontamination strategy to limit subsequent ESBL-E cross-transmission or infection, (6) all the patients were adults, and (7) they were written in English. If the studies lacked outcome data or provided only the prevalence of ESBL-E colonization or infection, they were excluded. If the full text could not be retrieved or if the article was a commentary or a review or an *erratum*, it was excluded.

### Selection of studies and data extraction

All the available data were extracted from each study by two investigators (DG and RP) independently according to the aforementioned inclusion criteria, and any differences were resolved by discussion with a third investigator (JRZ). The following data were collected from each study: the name of the first author, publication year, study design, number of patients, primary outcome, and risk factors for the primary outcome.

## Results

### Study selections

**Fig. 1:**
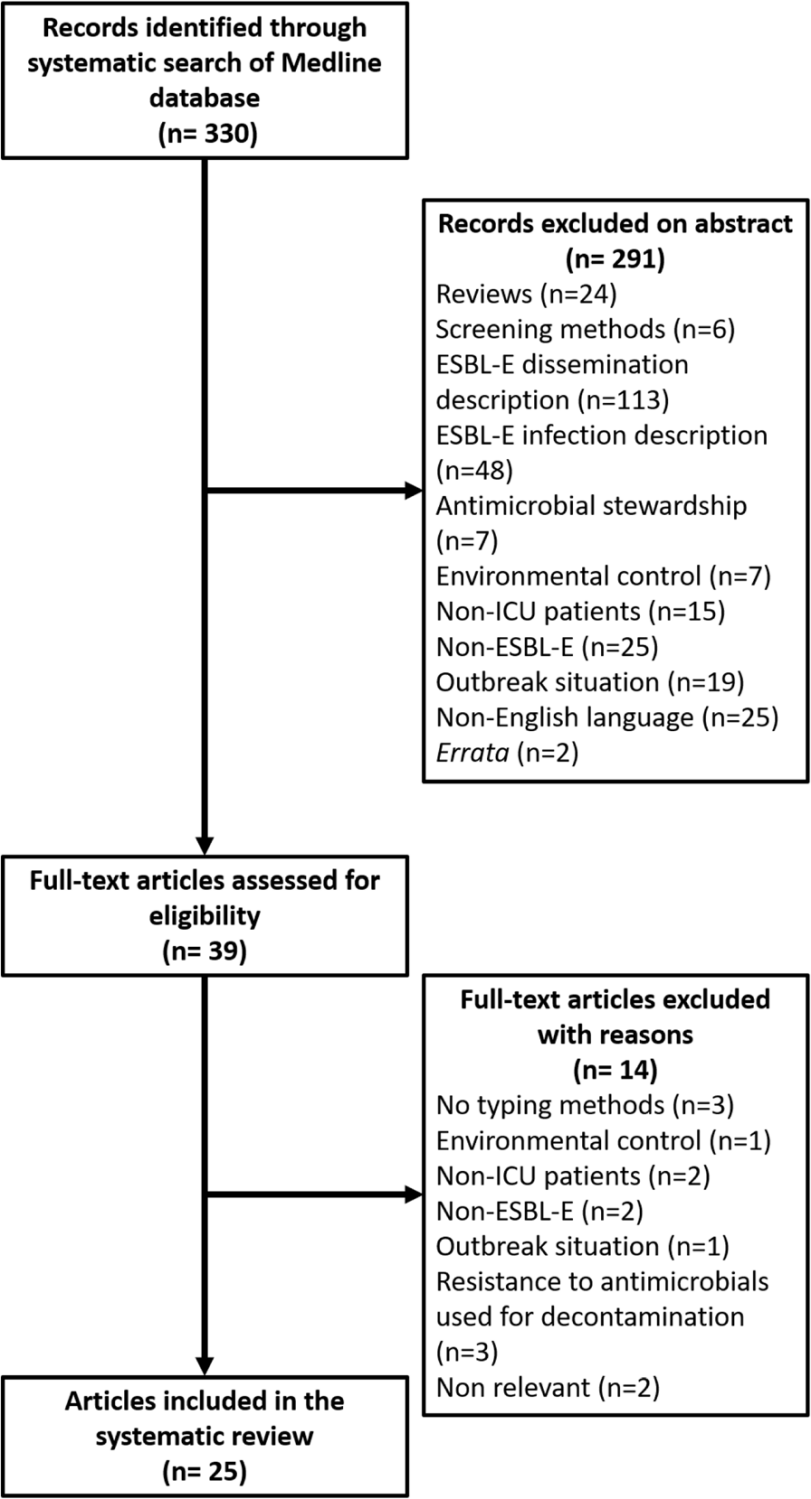
Flowchart of the study selection process

Using the previously described request in MEDLINE database, 330 articles were referenced. Every abstract was read and 39 articles that appeared to address issues relevant for this review were selected for full-text assessment. After full-text assessment, 25 studies were included in the systematic review (Fig. 1). Seven were relevant regarding the risk of ESBL-E cross-transmission (Table 1), 4 regarding the evaluation of hygiene procedures’ efficacy (Table 2), 10 assessed the link between colonization and infection or the prognostic value of ESBL-E carriage to guide the empirical antimicrobial therapy (Table 3), and 4 the efficacy of a selective decontamination strategy (SD) (Table 4).

**Table 1:** Low level of ICU ESBL-E cross-transmission in a non-outbreak situation

Year	Authors	Design	*N*	Outcome	Brief results
2017	Repessé et al. [28]	Cohort study	470	ESBL-E fecal carriage Cross-transmission assessed by epidemiology and ESBL gene sequencing	62/470 (13.2%) of imported ESBL-E fecal carriage 9/221 (4.1%) of acquired ESBL-E fecal carriage 2/9 acquisitions were likely to be due to cross-transmission
2016	Alves et al. [27]	Cohort study	309	ESBL-E fecal carriage Cross-transmission assessed by epidemiology, rep-PCR and plasmid PCR	25/309 (8%) of imported ESBL-E fecal carriage 19/309 (6.5%) of acquired ESBL-E fecal carriage 1/19 acquisition was likely to be due to cross-transmission
2015	O’Connell et al. [26]	Cohort study	316	ESBL-E fecal carriage Cross-transmission assessed by epidemiology and PFGE	50/316 (15.8%) of ESBL-E fecal carriage 2 cases of suspected cross-transmission for *E.coli* and 2 for *E. cloacae* but only 1 (*E. cloacae*) occurred in ICU
2014	Kim J et al. [25]	Cohort study	347	Acquisition of ESBL-E by epidemiology and PFGE	98/347 (28.2%) of imported ESBL-E fecal carriage 11/91 (12.1%) of acquired ESBL-E fecal carriage in ICU No case of cross-transmission
2007	Harris et al. [24]	Cohort study	1806	Acquisition of ESBL-producing *E. coli* by epidemiology and PFGE	97/1806 (5%) of ESBL-E fecal carriers including as follows: 23/97 (24%) of acquired ESBL-producing *E. coli* fecal carriage 3/23 (13%) acquisitions were likely to be due to cross-transmission
2004	Thouverez et al. [23]	Cohort study	2883	Acquisition of ESBL-E by epidemiology and PFGE	9/28 cases of ESBL-E acquisition explained by cross-transmission
1996	Gori et al. [22]	Cohort study	8640	Acquisition of ESBL-producing *K. pneumoniae* by antibiotype, plasmid content, PFGE, and RAPD	45/8640 (0.5%) ESBL-E fecal carriage 4 ESBL-producing *K. pneumoniae* clonal groups among which 2 are associated with clusters of cross-infection involving 5 and 12 patients

*ESBL-E* extended-spectrum beta-lactamase-producing *Enterobacteriaceae*, *ICU* intensive care unit, *PFGE* pulsed-field gel electrophoresis, *RAP* rapid amplified polymorphic DNA, *rep-PCR* repetitive-element polymerase chain reaction

**Table 2:** Efficacy of contact precautions on ICU ESBL-E dissemination in a non-outbreak situation

Year	Authors	Design	*N*	Outcome	Brief results
2018	Jalalzaï et al. [30]	Unicentric, retrospective, uncontrolled before-and-after study	524 SCP 545 non-SCP with SP	ICU-acquired ESBL-E infections ICU deaths	No independent impact on ESBL-E infections of cessation of admission screening (adjusted OR 1.16, 95% CI 0.38–3.50, *p* = 0.79) nor on in-ICU death (SHR 1.22, 95% CI 0.93–1.59, *p* = 0.15)
2017	Kardas-Stoma et al. [32]	Cost-effectiveness analysis	NA	ICU-acquired ESBL-E fecal carriage ICU-acquired ESBL-E infections	Universal screening and contact precautions for ESBL-E fecal carriers vs base care, per 100 admissions 12 vs 15 ICU-acquired ESBL-E fecal carriage 4 vs 5 ICU-acquired ESBL-E infections
2017	Renaudin et al. [31]	Prospective non-inferiority before-and-after study	1547 CP 1577 SP	ICU-acquired ESBL-E fecal carriage	Incidence densities respectively during CP and SP: 2.7 (95% CI 1.78–3.62), 2.06 (95% CI 1.27–2.86) per 1000 patient-days; *p* 0.004 for non-inferiority
2014	Derde et al. [29]	Prospective, randomized, interrupted, time series study	8501	ICU-acquired ESBL-E fecal carriage with and without CP	Incidence rate ratio: 0.994 (0.968–1.021; *p* 0.66) comparing with and without CP

*CP* contact precautions, *HH* hand hygiene, *ICU* intensive care unit, *PFGE* pulsed-field gel electrophoresis, *rep-PCR* repetitive-element Polymerase chain reaction, *SP* standard precautions, *SCP* screening period

**Table 3:** Evaluation of ESBL-E fecal carriage to tailor empirical antimicrobial therapy

Year	Authors	Design	*N*	Outcome	Brief results
2018	Jalalzaï et al. [30]	Monocentric, retrospective, before-and-after study	524 SCP 545 non-SCP	Carbapenem consumption	Decrease in carbapenem exposure in patients without ESBL-E infection during the non-SCP (75 vs 61 carbapenem-days per 1000 patient-days, *p* = 0.01)
2018	Barbier et al. [39]	Inception cohort of a multicenter prospective database	318	ESBL-E VAP	18 ESBL-E VAP for 361 (5%) ventilator-associated complications among ESBL-E fecal carriers
2018	Houard et al. [40]	Monocentric, retrospective cohort study	410	ESBL-E VAP	Previous ESBL-E fecal carriage as the only independent risk factor [OR 23; 95% CI (10–55), *p* < 0.001] Predictive value of ESBL-E fecal carriage for subsequent ESBL-E VAP: PPV 43.6%, NVP 97.3%
2018	Liu et al. [35]	Monocentric, retrospective nested case-control study	9015	ICU-acquired ESBL-E BSI	42 ESBL-E BSI among 9015 ESBL-E fecal carriers (0.5%) Independent risk factors associated with subsequent ESBL-E BSI: Antibiotic in the past 72 h: Penicillin (OR 12.076; 95% CI 1.397–104.251, *p* 0.024) Cephalosporin (OR 6.900; 95% CI 1.493–31.852, *p* 0.013) Carbapenem (OR 5.422; 95% CI 1.228–23.907, *p* 0.026) Previous ICU stay (OR 1.041; 95% CI 1.009–1.075, *p* 0,012) Maximum body temperature (OR 8.014; 95% CI 2.408–26.620, *p* 0.001)
2017	Razazi et al. [37]	Monocentric, prospective cohort study	6303	ICU-acquired ESBL-E pneumonia Predictive factors for ESBL-E pneumonia among carriers	48/843 (6%) ESBL-E fecal carriers has subsequent ICU-acquired ESBL-E pneumonia 48/111 (43%) of ICU-acquired pneumonia among ESBL-E fecal carriers were due to ESBL-E SAPSII at admission > 43 [OR 2.81 (1.16–6.79)] Colonization with *Enterobacter* sp. or *K. pneumoniae* [OR 10.96 (2.93–41.0)] Receipt of > 2 days of AMC [OR 0.24 (0.08–0.71)]
2017	Carbonne et al. [38]	Multicenter, retrospective cohort study	1503	ESBL-E pulmonary colonization	ESBL-E fecal carriage predictive values for ESBL-E pulmonary colonization: Early (≤ 5 days): NPV 99.2% (95% CI [98.7;99.6]), PPV 14.5% (95% CI [12.8;16.3]) Late (> 5 days): NPV 93.4% (95% CI [91.9;95.0]), PPV 34.4% (95% CI [31.4;37.4])
2016	Barbier et al. [41]	Cause-specific hazard model based on prospective data	16,374	ICU-acquired ESBL-E infection Carbapenem exposure	98/594 (16.4%) ESBL-E fecal carriers had subsequent ICU-acquired ESBL-E infection 627, 241 and 69 carbapenem-days per 1000 patient-days for respectively infected ESBL-E carriers, non-infected ESBL-E carriers and non ESBL-E carriers
2016	Bruyère et al. [19]	Monocentric, retrospective cohort study	587	ESBL-E VAP	ESBL-E fecal carriage predictive values for ESBL-E VAP: PPV 41.5%, NPV 99.4%
2012	Razazi et al. [34]	Monocentric, prospective, cohort study	610	ICU-acquired ESBL-E infection	10% of the first episodes of ICU-acquired infections are due to ESBL-E 27% of the second episodes of ICU-acquired infections are due to ESBL-E
2006	Martins et al. [36]	Monocentric prospective cohort study	231	ICU-acquired ESBL-producing *K. pneumoniae* pneumonia	Previous ESBL-production *K. pneumoniae* is an independent risk factor ICU-acquired ESBL-producing *K. pneumoniae* pneumonia (OR 60.6; 95% CI 56.33–578.73)

*AMC* amoxicillin/clavulanic acid, *BSI* bloodstream infection, *ESBL-E* extended-spectrum beta-lactamase-producing *Enterobacteriaceae*, *ICU* intensive care unit, *NPV* negative predictive value, *PPV* predictive positive value, *SCP* screening period, *SAPSII* Simplified Acute Physiology Score II, *VAP* ventilator-associated pneumonia

**Table 4:** Efficacy of selective decontamination for ESBL-E fecal carriage among ICU patients

Year	Authors	Design	Decontamination	*N*	Outcome	Brief results
2018	Wittekamp et al. [46]	Randomized controlled trial	CHX 2% SOD by mouthpaste (colistin, tobramycin, nystatin) SDD by the same mouthpaste and gastrointestinal suspension)	8665	ICU-acquired ESBL-E BSI	aHR vs baseline: CHX 1.13 (95% CI 0.68–1.88) SOD 0.89 (95% CI 0 .55–1.45) SDD 0.70 (95% CI 0.43–1.14)
2016	Camus et al. [44]	Observational Before-after	SDD by as follows: Colistin Tobramycin Amphotericin B	5250	Rates of acquired infections caused by AGNB Rates of ESBL-E fecal carriage acquisition	Diminution of the incidence rate of acquired infections caused by AGNB (1.59 vs 5.43 per 1000 patient-days, *p* < 0.001) Diminution of the acquisition rate of ESBL-E fecal carriage (OR = 0.94 [0.88–1.00], *p* = 0.04)
2005	Troché et al. [43]	Prospective observational cohort study	SDD by 2 among the following: Erythromycin Neomycin Polymyxin E	2235	Rates of ESBL-E fecal carriage acquisition	Diminution of the acquisition rate of ESBL-E fecal carriage from 5.5 cases per 1000 patient-days during the first 3 years to 1.9 cases during the last 3 years (*p* < 0.05)
1998	Decré et al. [45]	Prospective controlled cohort study	SDD by as follows: Erythromycin Polymyxin E	65	Incidence and infection with ESBL- *K. pneumoniae*	Selective digestive decolonization failed to reduce the incidence of acquisition of ESBL-producing *K. pneumoniae*

*AGNB* multidrug-resistant aerobic Gram-negative bacilli, *aHR* adjusted Hazard ratio, *BSI* bloodstream infection, *CHX* chlorhexidine, *SDD* selective digestive decontamination, *SOD* selective oral decontamination

### ESBL-E fecal carriage and the risk of cross-transmission in ICU in a non-outbreak situation

The first study demonstrating ICU ESBL-E cross-transmission in a non-outbreak situation describes two clones responsible for clusters of 5 and 12 patients. Nevertheless, this study included patient in 1990 and 1991 and did not focused on carriage but infection [22]. Another study involved 2883 patients with 28 (0.97%) ESBL-E carriers. Only 9/28 cases of ESBL-E were explained by cross-transmission despite the fact that the screening method was suboptimal limiting their impact on the prevention of ESBL-E dissemination [23]. These results were consistent with another one detecting 97/1806 (5%) of ESBL-E fecal carriers including 23/97 (24%) of ICU-acquired ESBL-producing *E. coli* fecal carriage with only 3/23 (13%) acquisitions likely to be due to cross-transmission [24]. Similar results were demonstrated even in a higher prevalence situation (ESBL-E carriage at admission: 98/347 (28.2%)), with an acquisition rate of 12.1% (11/91) without any case of cross-transmission [25]. Another study based on pulsed-field gel electrophoresis (PFGE), revealed two cases of suspected cross-transmission for *E. coli* and two for *E. cloacae* in a setting with 50/316 (15.8%) of ESBL-E fecal carriage from patients on the liver transplantation, ICU, and hematology/oncology wards. Nevertheless, only one case of suspected cross-transmission (*E. cloacae*) occurred in ICU [26]. In other studies, cross-transmission has also been shown to be a rare event (5% of ESBL-E acquisition) [27], even in an ICU with no single room [28]. The occurrence of ESBL-E acquisition despite limited cross-transmission suggests limits to hygiene procedure efficacy in controlling ESBL-E dissemination in a non-outbreak situation and other mechanisms of dissemination which we still have to investigate.

### ESBL-E fecal carriage and hygiene procedures in a non-outbreak situation

A large multicenter study in 13 European ICU testing the effect of rapid screening and isolation of carriers with contact precautions did not find any impact on ESBL-E acquisition, but the study was led in the context of a sustained high level of compliance to hand hygiene and chlorhexidine bathings [29]. In another study, cessation of contact isolation procedures had no independent impact on ESBL-E infections (adjusted OR 1.16, 95% CI 0.38–3.50, *p* = 0.79) nor on in-ICU death (SHR 1.22, 95% CI 0.93–1.59, *p* = 0.15) [30]. These results were consistent with a previous study which found that discontinuing contact precautions did not increase ICU-acquired ESBL-E fecal carriage (incidence densities respectively during contact vs standard precautions: 2.7 (95% CI 1.78–3.62) and 2.06 (95% CI 1.27–2.86) per 1000 patient-days; *p* 0.004 for non-inferiority) in an ICU with single rooms with dedicated equipment, strict application of hand hygiene, medical and paramedical leadership, and good antibiotic stewardship. [31].

A cost-effectiveness analysis showed that an improved compliance with hand hygiene is the most cost-saving strategy to prevent the transmission of ESBL-E. Screening and cohorting had comparable effectiveness but were more expensive; screening and contact precautions were the least effective strategy [32].

To summarize, these results together suggest that cross-transmission does not seem any more to be the main source of ESBL-E acquisition in ICU. Moreover, a universal standard precaution strategy seems to be sufficient to control the risk of cross-transmission. The relevance of a systematic ESBL-E fecal carriage screening policy in ICU to guide hygiene procedures is now questioned (Additional file 1).

### ESBL-E fecal carriage prognostic value for subsequent ICU-acquired ESBL-E infections in a non-outbreak situation

Another discussed interest of systematic screening of ESBL-E fecal carriage could be to guide empiric antimicrobial therapy in case of subsequent infection among carriers. The most investigated ICU-acquired infections are VAP and bloodstream infections (BSI) as they are the more frequent ones [33]. Among ESBL-E fecal carriers in ICU, one study found that 10% and 27% of first and second episodes of ICU-acquired infections [34] and another that even 40% of VAP are due to ESBL-E [19].

Regarding BSI, a recent study conducted in China found a proportion of 0.5% of ESBL-E fecal carriers developing subsequent ESBL-E BSI in ICU (42/9015). Independent risk factors associated with subsequent ESBL-E BSI were antibiotic use in the past 72 h (penicillin [OR 12.076; 95% CI 1.397–104.251, *p* 0.024], cephalosporin [OR 6.900; 95% CI 1.493–31.852, *p* 0.013], carbapenem [OR: 5422; 95% CI 1.228–23.907, *p* 0.026]), previous ICU stay (OR 1.041; 95% CI 1.009–1.075, *p* 0,012), and maximum body temperature (OR 8014; 95% CI 2.408–26.620, *p* 0.001) [35]. Nevertheless, as a case is diagnosed by a positive blood culture, antimicrobial therapy and maximum body temperature could only be the signal of the on-going infection with empiric treatment initiated as ESBL-E BSI are compared with non-infected ESBL-E fecal carriers.

Regarding risk factors for VAP, ESBL-producing *Klebsiella pneumoniae* was found to be an independent risk factor ICU-acquired ESBL-producing *K. pneumoniae* pneumonia (OR 60.6; 95% CI 56.33–578.73) [36]. Other identified independent risk factors for ESBL-E pneumonia among ESBL-E fecal carriers were SAPS II at admission > 43 [OR 2.81 (1.16–6.79)] and colonization with *Enterobacter* sp. or *K. pneumoniae* species [OR 10.96 (2.93–41.0)] whereas receipt of > 2 days of amoxicillin/clavulanic acid during the ICU stay was protective [OR 0.24 (0.08–0.71)] [37]. Despite these identified risk factors, individual prediction of subsequent ESBL-E VAP among ESBL-E fecal carriers remains difficult and prognostic values will be discussed thereafter.

Regarding ESBL-E pulmonary colonization, ESBL-E fecal carriage has an excellent negative predictive value (NPV) (99.2%, 95% CI [98.7,99.6] for ≤ 5 days and 93.4%, 95% CI [91.9,95.0] for > 5 days) despite a poor positive predictive value (PPV) (14.5% [95% CI 12.8, 16.3] and 34.4% [95% CI [31.4, 37.4]), for the early and late groups respectively [38].

Nevertheless, pulmonary colonization does not mean infection. A monocentric prospective study found 111 (13%) patients among 843 ESBL-E fecal carriers who developed ICU-acquired pneumonia of whom 48 (43%) had ESBL-E pneumonia (6% of carriers). Patients with ESBL-PE pneumonia in this study had a higher SOFA score (*p* = 0.037) and more frequent septic shock at pneumonia onset (*p* = 0.047) than patients with pneumonia due to another germ [37]. Even if this article also identified risk factors, it remains very difficult to predict which patient will suffer from a ESBL-E VAP or not. Enhancing this result, an inception cohort of the prospective database OUTCOMEREA showed that infectious-related ventilator-associated complications among ESBL-E fecal carriers mostly reflect non-VAP events (18/361, 13%) with only 18/361 (5%) ESBL-E VAP but that they are a major driver of carbapenem consumption [39]. Regarding predictive values, a retrospective monocentric study showed a 41.5% PPV and a 99.4% NPV [19]. Another study confirmed prior ESBL-E fecal carriage to be the only independent risk factor for subsequent ESBL-E VAP [OR 23 (95% CI 10–55)] with a PPV of 43.6% and a NPV of 97.3%. Duration of mechanical ventilation, length of ICU stays, and mortality rates (55.8% vs 50%, *p* = 0.48) were similar in ESBL-E VAP, compared with VAP due to other bacteria [40]. Because of these excellent NPV, some authors have suggested that systematic ESBL-E fecal carriage screening could help to limit the use of carbapenems, but this assertion is now contradicted.

Jalalzaï et al. assessed carbapenem consumption after cessation of screening for intestinal carriage of ESBL-E during two consecutive 1-year period (with and without systematic screening with respectively 524 and 545 patients) [30]. An admission during the no-systematic screening period exerted no independent impact on the hazards of ESBL-E infections and in-ICU death. The exposure to carbapenems in patients without ESBL-E infection even decreased between the systematic screening and no-systematic screening periods (75 versus 61 carbapenem-days per 1000 patient-days, *p* = 0.01). These results are consistent with a prospective multicentric study revealing that ESBL-E infections are rather infrequent in carriers and that carbapenem exposure was increased among ESBL-E carriers without infection. ESBL-E carriers even without infections also had a delayed discharge, thereby amplifying the selective pressure and the colonization pressure in ICU [41].

To summarize, the link between ESBL-E colonization and subsequent ESBL-E infection seems to be real as consistently observed by several different teams. Nevertheless, subsequent ESBL-E infections are a rare event which is almost unpredictable. An empirical antimicrobial therapy guided on ESBL-E carriage status leads to an overconsumption of carbapenems without a clinical benefit. A systematic screening policy of ESBL-E fecal carriage in ICU to guide empirical antimicrobial therapy is now questioned. Further studies are needed to better understand the link between colonization and infection and to assess if we can improve the prediction of subsequent ESBL-E infections. Disturbances of gut microbiota could be part of the explanation, and so selective decontamination has been evoked as a tool to modulate the gut microbiota and to eradicate multi-drug resistant bacteria fecal carriage [21, 42].

#### ESBL-E fecal carriage and selective decontamination in ICU in a non-outbreak situation

One prospective observational cohort study in a surgical ICU of a tertiary teaching hospital using 2 antibiotics among erythromycin, neomycin, or polymyxin E in 37 ESBL-E fecal carriers (2235 patients included) suggested a diminution of the acquisition rate of ESBL-E fecal carriage from 5.5 cases per 1000 patient-days during the first 3 years to 1.9 cases during the last 3 years (*p* < 0.05) but it was a secondary outcome [43]. Additionally, an observational single-center study found a diminution of the incidence rate of acquired infections caused by multidrug-resistant aerobic Gram-negative bacilli (1.59 vs 5.43 per 1000 patient-days, *p* < 0.001) and a diminution of the acquisition rate of ESBL-E fecal carriage (OR = 0.94 [0.88–1.00], *p* = 0.04) [44]. Another prospective controlled cohort study with decolonization by erythromycin and polymyxin E concluded that selective digestive decolonization (SDD) failed to reduce the incidence of acquisition of ESBL-producing *K. pneumoniae* [45]. Nevertheless, these studies did not assess the efficacy of SDD especially for the ESBL-E carriers. A recent multicenter prospective study including 8665 patients compared the efficacy of chlorhexidine 2% vs selective oral decontamination (SOD) by mouthpaste (colistin, tobramycin, nystatin) vs SDD by the same mouthpaste and gastrointestinal suspension to prevent ESBL-E bloodstream infections but among every ventilated patients in ICU and not only ESBL-E carriers. Compared to baseline care, they did not find any strategy to be efficient (adjusted hazard ratios: CHX 1.13 (95% CI 0.68–1.88), SOD 0.89 (95% CI 0.55–1.45), SDD 0.70 (95% CI 0.43–1.14) [46].

Because of the lack of high-quality evidence of selective decontamination efficacy (oral and/or digestive) and concerns about the emergence of resistance to the antimicrobials used, European guidelines recommend against decolonization strategies for ICU ESBL-E fecal carriers and call for more research [47]. According to current knowledge, it does not seem relevant to have a systematic ESBL-E fecal carriage screening policy to guide selective decontamination strategy as ESBL-E gut decolonization by antibiotics are not validated and so not recommended. Nevertheless, the impact of SDD on ESBL carriage was only evaluated in studies performed in western countries. This field seems to be helpful and should be better explored.

## Discussion

A universal standard precaution strategy seems to be sufficient to control the risk of ESBL-E cross-transmission even in ICU. A systematic screening policy to guide contact precautions does not appear to be useful and cost-effective. Therefore, it should be kept in mind that the studies reported here assessed a non-outbreak situation in western countries. A study assessing the ESBL-E respiratory colonization in Sri Lanka suggested a higher cross-transmission rate but no data are provided about the imported or acquired status of ESBL-E colonization [48]. Further studies are needed to assess the rate of cross-transmission and the efficacy of hygiene procedures in non-western countries which have a higher prevalence of ESBL-E [2, 16]. Moreover, the four studies presented in Table 2 have some methodological limitations even if they all draw the same conclusions without any increase in ESBL-E infections after cessation of contact precautions for strict standard precautions and that studies conducted in a non-ICU setting discussed thereafter conclude the same way.

Regarding the efficacy of hygiene procedures in non-ICU setting, a quasi-experimental study showed that contact precautions prevent from outbreaks but have no impact on nosocomial ESBL incidence in a non-outbreak situation [49]. Then, cessation of contact precautions for ESBL-producing *E.coli* was first demonstrated to be safe among non-ICU hospitalized patients in double-bed rooms with another patient colonized or infected with an ESBL-E [50] and was confirmed by the absence of cross-transmission with the respect of the sole standard hygiene precautions [51]. In addition, a study showed no difference between standard and contact precautions in the incidence of ESBL-E in hospital settings [52]. Besides the lack of efficacy, contact precautions are associated with adverse effects including patients’ psychological distress or medical errors [53, 54]. Another point is that even if cross-transmission can be a cause of ESBL-E carriage acquisition, it remains a rare event in western countries ICU under strict hygiene procedures. For instance, even in a study with a large proportion of cross-transmission among ICU-acquired ESBL-E carriage acquisition (3/23, 13%), in fact only 3/1806 (0.17%) patients admitted in ICU during the study period experienced ESBL-E cross-transmission [24]. Moreover, even one case of cross-transmission will always be concerning and thorough standard procedures are absolutely needed.

Besides, the ability for spreading of the different species of *Enterobacteriaceae* could be variable with a special concern for *K pneumoniae*. Some authors suggest it could be 3.7 times more prone to cross-transmission than *E.coli* [55]. Nevertheless, the mathematical model assuming a 100% sensitivity and specificity for microbiological tests and the absence of interaction between *E. coli* and non-*E. coli* bacteria cannot be fully realistic and so limit the validity of these results. It remains also unclear if this difference of transmissibility relies on bacteria intrinsic virulence or patients’ frailty. Colonization seems to occur in patients with many comorbidities, invasive procedures, and antimicrobial exposure, who have a higher colonizing inoculum leading to a possible increased risk of cross-transmission [56, 57]. Furthermore, cross-transmission was a rare event even for *K. pneumoniae* as reported previously [27, 28]. No data prove any difference between standard and contact precautions regarding *K. pneumoniae* cross-transmission [58].

Targeted screening was also studied because the risk of ESBL-E fecal carriage is not equal for every in-patient in ICU [59]. This targeted strategy has been confirmed to be as efficient as the systematic one with exposure to antibiotics within the preceding 3 months, hospitalization within the preceding year, admission of another hospital department with a hospital stay of more than 5 days, immunosuppression, chronic dialysis, transfer from rehabilitation, long-term-care unit or nursing home, and travel abroad within 1 year as targeted risk factors. Results were consistent with those of another study which considered transfer from another unit or hospital as risk factors and did not find any association with more third-generation cephalosporin (3CG)-resistant infections (ESBL or production of cephalosporinase) [60]. Nevertheless, the targeted strategy has not been compared to the complete cessation of screening and there is so no proof of its relevance.

Even if the relevance of universal screening and of contact precautions is questioned, it should not be interpreted as a lax signal. Once again, high compliance with systematic standard precautions, especially hand hygiene, permanent surveillance of nosocomial ESBL-E infection outbreak, and antimicrobial stewardship are fully needed [61].

### Treatment of subsequent infections

Several studies aimed to identify reliable risk factors to predict subsequent ESBL-E infections. In non-ICU patients, severity at admission and colonization with *Enterobacter* sp. or *K. pneumoniae*; referral from a medical ward, nursing home, or rehabilitation center; previous fluoroquinolone treatment; extracorporeal membrane oxygenation; and the absence of prior positive ESBL-E rectal swab culture were identified as risk factors [62]. Despite these identified risk factors, it remains difficult to predict which patient is infected with an ESBL-E or not, especially in ICU [39]. The effect of BLI use prior to infection among ESBL-E fecal carriers remains unclear [37, 63]. Available data focus on VAP and BSI as they are the most frequent ICU-acquired infections and as the distinction between urinary tract infection and urinary colonization in catheterized patients can be seriously challenging.

As described for the risk of transmission, *K. pneumoniae* could be at increased risk of subsequent infections in colonized patients compared with other *Enterobacteriaceae* [55, 56, 64]. Moreover, no significant difference in hospital mortality in non-ICU patients has been found between *E. coli* and *K. pneumoniae* (ESBL *E. coli* 23.8% vs ESBL *K. pneumoniae* 27.1%, *p* = 0.724) [65]. Once again, the role of the bacteria itself or of the host’s condition is still unclear [56]. ESBL-E carriage, *a fortiori* for *K. pneumoniae*, could be a major reflection of the host frailty. The fact that adequate empirical therapy does not have a clear impact on ESBL-E infections contrary to the host’s condition supports this hypothesis [66–69]. The more recent study on the field is the only randomized controlled trial available and did not manage to prove the non-inferiority of piperacillin-tazobactam compared with meropenem for the treatment of BSI due to 3CG-resistant *E.coli* or *K. pneumoniae* [69]. Nevertheless, despite the quality of this study, some limitations apply to the conclusions of this study which should be interpreted cautiously [70]. Further studies are urgently needed to address this issue. Therefore, systematic carbapenem use among subsequently infected ESBL-E carriers could be questioned. In that case, main alternative candidates would be combinations such as piperacillin-tazobactam, new beta-lactams as ceftolozane-tazobactam or ceftazidime-avibactam, and combination with aminoglycosides [71–74]. Those strategies need to be validated but would allow carbapenems spare without the need of systematic ESBL-E fecal carriage screening.

### Selective decontamination

Selective decontamination is an attractive approach but, as previously described, its efficacy for ESBL-E decontamination still needs to be proven. Moreover, some concerns are already raised about the emergence of resistance to used antimicrobials such as colistin and tobramycin [75–77]. One of these studies even showed the emergence of ESBL-E when 3CG are used for selective decontamination [77]. Regarding a non-ICU setting, the major study was a single-center trial with 54 ESBL-E carriers involved which assessed colistin and neomycin plus nitrofurantoin to successfully decolonize patients during the treatment but not 7 days after treatment cessation. Furthermore, no clinical outcome was reported [78].

Another approach to eradicate ESBL-E fecal carriage would be to cope with the effects of critical illness and antimicrobial therapy on gut. In fact, major gut microbiota modifications occur during hospitalization, *a fortiori* in ICU, and some of these modifications are associated ESBL-E fecal carriage [79, 80]. Cases of FMT to eradicate ESBL-E carriage have been reported [81, 82]. This approach is now being investigated in non-ICU patients. A proof-of-principle study included 15 patients to receive one FMT (among whom 7 received a second one) and suggested that FMT could be an effective treatment in patients carrying ESBL-E with several possible factors of response to therapy, such as donor-recipient microbiota match and number of FMTs [83]. A randomized, open-label, superiority trial in four tertiary care centers showed a non-significant decolonization success of 1.7 [95% CI 0.4–6.4] for a 5-day course of oral antibiotics followed by FMT, but the study failed to achieve the planned sample size for logistical and regulatory reasons making firm conclusions regarding efficacy difficult [84]. Moreover, fecal transplantation is not always well accepted by the patients and their siblings and concerns exist about gut permeability among critically ill patients [85]. A “soft decolonization” approach by probiotics could be a more feasible option. Nevertheless, this field has to be considered with caution and remains controversial, since there are studies with some positive effects, but also many negative studies. A major limit of previous studies is that used probiotics were “ready-to-use.” A promising but still experimental approach is to tailor the probiotics by indication or even by patient. A first step forward is the identification of a four-bacteria consortium which eradicates gut colonization with vancomycin-resistant *Enterococcus faecium* in a murine model [86]. Regarding ESBL-E colonization, a recent study identified *Bacteroïdes uniformis* as a possible candidate [87]. FMT and “soft decolonization” by tailored probiotics have to be further investigated and shown to be effective and safe among ICU patients before it can be used in routine. Therefore, systematic ESBL-E fecal carriage screening cannot be indicated to guide non-validated therapies.

## Conclusions

Systematic ESBL-E fecal carriage screening in ICU and contact precautions have been set up to fight against ESBL-E outbreaks. In front of a changing paradigm from clonal outbreaks in health settings to a wide dissemination in the community, systematic ESBL-E screening could not be adequate anymore to guide contact precautions. Systematic and thorough standard precautions with hand hygiene appear to be the most efficient procedures even if studies providing higher level of evidence are warranted to strongly recommend in favor of or against systematic ESBL-E fecal carriage screening in ICU to guide hygiene procedures. Systematic ESBL-E fecal carriage screening seems to increase the consumption of carbapenems without improving the patients’ care and so does not seem to be beneficial as the overuse of carbapenems is associated with the expansion of carbapenem-resistant *Enterobacteriaceae*. The link between ESBL-E colonization and infection should be further investigated to better understand involved mechanisms. Selective decontamination could be a helpful approach to eradicate ESBL-E colonization, but its efficacy still needs to be demonstrated and concerns exist about the emergence of resistance to the antimicrobials used. New strategies for ESBL-E eradication modulating the gut microbiota such as FMT or a “soft decolonization” approach should be considered in future studies.

## Additional file

Additional file 1: **Figure S1.** Factors questioning the relevance of systematic fecal carriage screening of ESBL-E. ESBL-E: extended-spectrum beta-lactamase-producing *Enterobacteriaceae*. ICU: intensive care unit. (DOCX 98 kb)

## Abbreviations

3CG: Third-generation cephalosporins
BLI: Beta-lactam/beta-lactamase inhibitor
BSI: Bloodstream infection
CTX-M: CefoTaXimase-München
*E. coli*: *Escherichia coli*
ESBL-E: Extended-spectrum beta-lactamase-producing *Enterobacteriaceae*
FMT: Fecal microbiota transplantation
ICU: Intensive care unit
*K. pneumoniae*: *Klebsiella pneumoniae*
NPV: Negative predictive value
PCR: Polymerase chain reaction
PFGE: Pulsed-field gel electrophoresis
PPV: Positive predictive value
SDD: Selective digestive decontamination
SHV: SulpHydryl variable
SOD: Selective oral decontamination
VAP: Ventilator-associated pneumonia

## References

[1] Carlet J (2015). The world alliance against antibiotic resistance: consensus for a declaration. Clin Infect Dis.

[2] Woerther P-L, Burdet C, Chachaty E, Andremont A (2013). Trends in human fecal carriage of extended-spectrum -lactamases in the community: toward the globalization of CTX-M. Clin Microbiol Rev.

[3] Flokas ME, Alevizakos M, Shehadeh F, Andreatos N, Mylonakis E (2017). Extended-spectrum β-lactamase-producing Enterobacteriaceae colonisation in long-term care facilities: a systematic review and meta-analysis. Int J Antimicrob Agents.

[4] Nicolas-Chanoine M-H, Gruson C, Bialek-Davenet S, Bertrand X, Thomas-Jean F, Bert F (2013). 10-fold increase (2006-11) in the rate of healthy subjects with extended-spectrum -lactamase-producing Escherichia coli faecal carriage in a Parisian check-up centre. J Antimicrob Chemother.

[5] Tansarli GS, Karageorgopoulos DE, Kapaskelis A, Falagas ME (2013). Impact of antimicrobial multidrug resistance on inpatient care cost: an evaluation of the evidence. Expert Rev Anti-Infect Ther.

[6] Maslikowska JA, Walker SAN, Elligsen M, Mittmann N, Palmay L, Daneman N (2016). Impact of infection with extended-spectrum β-lactamase-producing Escherichia coli or Klebsiella species on outcome and hospitalization costs. J Hosp Infect.

[7] Esteve-Palau E, Solande G, Sánchez F, Sorlí L, Montero M, Güerri R (2015). Clinical and economic impact of urinary tract infections caused by ESBL-producing Escherichia coli requiring hospitalization: a matched cohort study. J Inf Secur.

[8] Leistner R, Gürntke S, Sakellariou C, Denkel LA, Bloch A, Gastmeier P (2014). Bloodstream infection due to extended-spectrum beta-lactamase (ESBL)-positive K. pneumoniae and E. coli: an analysis of the disease burden in a large cohort. Infection.

[9] Tacconelli E, Cataldo MA, Dancer SJ, De Angelis G, Falcone M, Frank U (2014). ESCMID guidelines for the management of the infection control measures to reduce transmission of multidrug-resistant Gram-negative bacteria in hospitalized patients. Clin Microbiol Infect.

[10] March A, Aschbacher R, Dhanji H, Livermore DM, Böttcher A, Sleghel F (2010). Colonization of residents and staff of a long-term-care facility and adjacent acute-care hospital geriatric unit by multiresistant bacteria. Clin Microbiol Infect.

[11] Price LB, Johnson JR, Aziz M, Clabots C, Johnston B, Tchesnokova V, et al. The epidemic of extended-spectrum-lactamase-producing *Escherichia coli* ST131 is driven by a single highly pathogenic subclone, H30-Rx. mBio. 2013;4 Available from: http://mbio.asm.org/cgi/doi/10.1128/mBio.00377-13. [cited 2018 Oct 31]. 10.1128/mBio.00377-13 PMC3870262 24345742

[12] Bauernfeind A, Grimm H, Schweighart S (1990). A new plasmidic cefotaximase in a clinical isolate of Escherichia coli. Infection.

[13] Siegel JD, Rhinehart E, Jackson M, Chiarello L (2007). 2007 guideline for isolation precautions: preventing transmission of infectious agents in health care settings. Am J Infect Control.

[14] Lowe CF, Katz K, McGeer AJ, Muller MP (2013). For the Toronto ESBL working group. Efficacy of admission screening for extended-spectrum beta-lactamase producing Enterobacteriaceae. Kluytmans J, editor. PLoS One.

[15] Société française d’hygiène hospitalière (SF2H) (2009). Prévention de la transmission croisée: précations complémentaires contact.

[16] Karanika S, Karantanos T, Arvanitis M, Grigoras C, Mylonakis E (2016). Fecal colonization with extended-spectrum beta-lactamase–producing *Enterobacteriaceae* and risk factors among healthy individuals: a systematic review and metaanalysis. Clin Infect Dis.

[17] Armand-Lefèvre L, Angebault C, Barbier F, Hamelet E, Defrance G, Ruppé E (2013). Emergence of imipenem-resistant Gram-negative bacilli in intestinal flora of intensive care patients. Antimicrob Agents Chemother.

[18] McLaughlin M, Advincula MR, Malczynski M, Qi C, Bolon M, Scheetz MH (2013). Correlations of antibiotic use and carbapenem resistance in Enterobacteriaceae. Antimicrob Agents Chemother.

[19] Bruyère R, Vigneron C, Bador J, Aho S, Toitot A, Quenot J-P, et al. Significance of prior digestive colonization with extended-spectrum β-lactamase–producing Enterobacteriaceae in patients with ventilator-associated pneumonia. Crit Care Med. 2016;44(4):699–706. 10.1097/CCM.0000000000001471 26571186

[20] Bretonnière C, Leone M, Milési C, Allaouchiche B, Armand-Lefevre L, Baldesi O (2015). Strategies to reduce curative antibiotic therapy in intensive care units (adult and paediatric). Intensive Care Med.

[21] Carlet J (2012). The gut is the epicentre of antibiotic resistance. Antimicrob Resist Infect Control.

[22] Gori A, Espinasse F, Deplano A, Nonhoff C, Nicolas MH, Struelens MJ (1996). Comparison of pulsed-field gel electrophoresis and randomly amplified DNA polymorphism analysis for typing extended-spectrum-beta-lactamase-producing Klebsiella pneumoniae. J Clin Microbiol.

[23] Thouverez M, Talon D, Bertrand X (2004). Control of Enterobacteriaceae producing extended-spectrum beta-lactamase in intensive care units: rectal screening may not be needed in non-epidemic situations. Infect Control Hosp Epidemiol.

[24] Harris AD, Kotetishvili M, Shurland S, Johnson JA, Morris JG, Nemoy LL (2007). How important is patient-to-patient transmission in extended-spectrum β-lactamase Escherichia coli acquisition. Am J Infect Control.

[25] Kim J, Lee JY, Kim SI, Song W, Kim J-S, Jung S (2014). Rates of fecal transmission of extended-spectrum β-lactamase-producing and carbapenem-resistant Enterobacteriaceae among patients in intensive care units in Korea. Ann Lab Med.

[26] O’Connell N, Keating D, Kavanagh J, Schaffer K (2015). Detection and characterization of extended-spectrum beta-lactamase-producing Enterobacteriaceae in high-risk patients in an Irish tertiary care hospital. J Hosp Infect.

[27] Alves M, Lemire A, Decré D, Margetis D, Bigé N, Pichereau C, et al. Extended-spectrum beta-lactamase − producing enterobacteriaceae in the intensive care unit: acquisition does not mean cross-transmission. BMC Infect Dis. 2016;16 Available from: http://bmcinfectdis.biomedcentral.com/articles/10.1186/s12879-016-1489-z. [cited 2018 Oct 31]. 10.1186/s12879-016-1489-z PMC4831109 27075040

[28] Repessé X, Artiguenave M, Paktoris-Papine S, Espinasse F, Dinh A, Charron C, et al. Epidemiology of extended-spectrum beta-lactamase-producing Enterobacteriaceae in an intensive care unit with no single rooms. Ann Intensive Care. 2017;7 Available from: http://annalsofintensivecare.springeropen.com/articles/10.1186/s13613-017-0295-0. [cited 2018 Oct 31]. 10.1186/s13613-017-0295-0 PMC5495817 28674848

[29] Derde LPG, Cooper BS, Goossens H, Malhotra-Kumar S, Willems RJL, Gniadkowski M (2014). Interventions to reduce colonisation and transmission of antimicrobial-resistant bacteria in intensive care units: an interrupted time series study and cluster randomised trial. Lancet Infect Dis.

[30] Jalalzaï W, Boutrot M, Guinard J, Guigon A, Bret L, Poisson D-M (2018). Cessation of screening for intestinal carriage of extended-spectrum β-lactamase-producing Enterobacteriaceae in a low-endemicity intensive care unit with universal contact precautions. Clin Microbiol Infect.

[31] Renaudin L, Llorens M, Goetz C, Gette S, Citro V, Poulain S (2017). Impact of discontinuing contact precautions for MRSA and ESBLE in an intensive care unit: a prospective noninferiority before and after study. Infect Control Hosp Epidemiol.

[32] Kardaś-Słoma L, Lucet J-C, Perozziello A, Pelat C, Birgand G, Ruppé E (2017). Universal or targeted approach to prevent the transmission of extended-spectrum beta-lactamase-producing Enterobacteriaceae in intensive care units: a cost-effectiveness analysis. BMJ Open.

[33] Lepape A, Machut A, Savey A (2018). Réseau national Réa-Raisin de surveillance des infections acquises en réanimation adulte - Méthodes et principaux résultats. Méd Intensive Réanimation.

[34] Razazi K, Derde LPG, Verachten M, Legrand P, Lesprit P, Brun-Buisson C (2012). Clinical impact and risk factors for colonization with extended-spectrum β-lactamase-producing bacteria in the intensive care unit. Intensive Care Med.

[35] Liu M, Li M, Wu L, Song Q, Zhao D, Chen Z (2018). Extended-spectrum β-lactamase-producing *E. coli* septicemia among rectal carriers in the ICU: Medicine (Baltimore).

[36] Martins IS, Pessoa-Silva CL, Nouer SA, Pessoa de Araujo EG, Ferreira ALP, Riley LW (2006). Endemic extended-spectrum beta-lactamase-producing Klebsiella pneumoniae at an intensive care unit: risk factors for colonization and infection. Microb Drug Resist.

[37] Razazi K, Mekontso Dessap A, Carteaux G, Jansen C, Decousser J-W, de Prost N, et al. Frequency, associated factors and outcome of multi-drug-resistant intensive care unit-acquired pneumonia among patients colonized with extended-spectrum β-lactamase-producing Enterobacteriaceae. Ann Intensive Care. 2017;7 Available from: http://annalsofintensivecare.springeropen.com/articles/10.1186/s13613-017-0283-4. [cited 2018 Oct 31]. 10.1186/s13613-017-0283-4 PMC5468364 28608133

[38] Carbonne H, Le Dorze M, Bourrel A-S, Poupet H, Poyart C, Cambau E, et al. Relation between presence of extended-spectrum β-lactamase-producing Enterobacteriaceae in systematic rectal swabs and respiratory tract specimens in ICU patients. Ann Intensive Care. 2017;7 Available from: http://annalsofintensivecare.springeropen.com/articles/10.1186/s13613-017-0237-x. [cited 2018 Oct 31]. 10.1186/s13613-017-0237-x PMC5289933 28155050

[39] Barbier F, Bailly S, Schwebel C, Papazian L, Azoulay É, for the OUTCOMEREA Study Group (2018). Infection-related ventilator-associated complications in ICU patients colonised with extended-spectrum β-lactamase-producing Enterobacteriaceae. Intensive Care Med.

[40] Houard M, Rouzé A, Ledoux G, Six S, Jaillette E, Poissy J (2018). Relationship between digestive tract colonization and subsequent ventilator-associated pneumonia related to ESBL-producing Enterobacteriaceae. Kou YR, editor. PLoS One.

[41] Barbier F, Pommier C, Essaied W, Garrouste-Orgeas M, Schwebel C, Ruckly S (2016). Colonization and infection with extended-spectrum β-lactamase-producing Enterobacteriaceae in ICU patients: what impact on outcomes and carbapenem exposure?. J Antimicrob Chemother.

[42] Brun-Buisson C, Legrand P, Rauss A, Richard C, Montravers F, Besbes M (1989). Intestinal decontamination for control of nosocomial multiresistant gram-negative bacilli. Study of an outbreak in an intensive care unit. Ann Intern Med.

[43] Troché G, Joly L-M, Guibert M, Zazzo J-F (2005). Detection and treatment of antibiotic-resistant bacterial carriage in a surgical intensive care unit: a 6-year prospective survey. Infect Control Hosp Epidemiol.

[44] Camus C, Sauvadet E, Tavenard A, Piau C, Uhel F, Bouju P (2016). Decline of multidrug-resistant Gram negative infections with the routine use of a multiple decontamination regimen in ICU. J Inf Secur.

[45] Decré D, Gachot B, Lucet JC, Arlet G, Bergogne-Bérézin E, Régnier B (1998). Clinical and bacteriologic epidemiology of extended-spectrum beta-lactamase-producing strains of Klebsiella pneumoniae in a medical intensive care unit. Clin Infect Dis.

[46] Wittekamp BH, Plantinga NL, Cooper BS, Lopez-Contreras J, Coll P, Mancebo J (2018). Decontamination strategies and bloodstream infections with antibiotic-resistant microorganisms in ventilated patients: a randomized clinical trial. JAMA.

[47] Tacconelli E (2018). EUCIC medical guidelines on decolonization of multidrug-resistant gram-negative organisms.

[48] Tissera K, Liyanapathirana V, Dissanayake N, Pinto V, Ekanayake A, Tennakoon M (2017). Spread of resistant gram negatives in a Sri Lankan intensive care unit. BMC Infect Dis.

[49] Conterno LO, Shymanski J, Ramotar K, Toye B, Zvonar R, Roth V (2007). Impact and cost of infection control measures to reduce nosocomial transmission of extended-spectrum β-lactamase-producing organisms in a non-outbreak setting. J Hosp Infect.

[50] Tschudin-Sutter S, Frei R, Dangel M, Stranden A, Widmer AF (2012). Rate of transmission of extended-spectrum beta-lactamase-producing Enterobacteriaceae without contact isolation. Clin Infect Dis.

[51] Souverein D, Euser SM, Herpers BL, Hattink C, Houtman P, Popma A, et al. No nosocomial transmission under standard hygiene precautions in short term contact patients in case of an unexpected ESBL or Q&A *E. coli* positive patient: a one-year prospective cohort study within three regional hospitals. Antimicrob Resist Infect Control. 2017;6 Available from: http://aricjournal.biomedcentral.com/articles/10.1186/s13756-017-0228-6. [cited 2018 Oct 31]. 10.1186/s13756-017-0228-6 PMC5485576 28670449

[52] Zahar J-R, Poirel L, Dupont C, Fortineau N, Nassif X, Nordmann P. About the usefulness of contact precautions for carriers of extended-spectrum beta-lactamase-producing Escherichia coli. BMC Infect Dis. 2015;15 Available from: http://bmcinfectdis.biomedcentral.com/articles/10.1186/s12879-015-1244-x. [cited 2018 Oct 31]. 10.1186/s12879-015-1244-x PMC4642679 26563141

[53] Zahar JR, Garrouste-Orgeas M, Vesin A, Schwebel C, Bonadona A, Philippart F (2013). Impact of contact isolation for multidrug-resistant organisms on the occurrence of medical errors and adverse events. Intensive Care Med.

[54] Morgan DJ, Diekema DJ, Sepkowitz K, Perencevich EN (2009). Adverse outcomes associated with contact precautions: a review of the literature. Am J Infect Control.

[55] Gurieva T, Dautzenberg MJD, Gniadkowski M, Derde LPG, Bonten MJM, Bootsma MCJ (2018). The transmissibility of antibiotic-resistant Enterobacteriaceae in intensive care units. Clin Infect Dis.

[56] Scheuerman O, Schechner V, Carmeli Y, Gutiérrez-Gutiérrez B, Calbo E, Almirante B (2018). Comparison of predictors and mortality between bloodstream infections caused by ESBL-producing Escherichia coli and ESBL-producing Klebsiella pneumoniae. Infect Control Hosp Epidemiol.

[57] Freeman JT, Rubin J, McAuliffe GN, Peirano G, Roberts SA, Drinković D (2014). Differences in risk-factor profiles between patients with ESBL-producing Escherichia coli and Klebsiella pneumoniae: a multicentre case-case comparison study. Antimicrob Resist Infect Control.

[58] Han JH, Bilker WB, Nachamkin I, Zaoutis TE, Coffin SE, Linkin DR (2013). The effect of a hospital-wide urine culture screening intervention on the incidence of extended-spectrum β-lactamase-producing Escherichia coli and Klebsiella species. Infect Control Hosp Epidemiol.

[59] Djibré M, Fedun S, Le Guen P, Vimont S, Hafiani M, Fulgencio J-P (2017). Universal versus targeted additional contact precautions for multidrug-resistant organism carriage for patients admitted to an intensive care unit. Am J Infect Control.

[60] Dananché C, Bénet T, Allaouchiche B, Hernu R, Argaud L, Dauwalder O (2015). Targeted screening for third-generation cephalosporin-resistant Enterobacteriaceae carriage among patients admitted to intensive care units: a quasi-experimental study. Crit Care.

[61] Tschudin-Sutter S, Lucet J-C, Mutters NT, Tacconelli E, Zahar JR, Harbarth S (2017). Contact precautions for preventing nosocomial transmission of extended-spectrum β lactamase–producing Escherichia coli: a point/counterpoint review. Clin Infect Dis.

[62] Vodovar D, Marcadé G, Rousseau H, Raskine L, Vicaut E, Deye N (2014). Predictive factors for extended-spectrum beta-lactamase producing Enterobacteriaceae causing infection among intensive care unit patients with prior colonization. Infection.

[63] Goulenok T, Ferroni A, Bille E, Lécuyer H, Join-Lambert O, Descamps P (2013). Risk factors for developing ESBL E. coli: can clinicians predict infection in patients with prior colonization?. J Hosp Infect.

[64] Gorrie CL, Mirčeta M, Wick RR, Edwards DJ, Thomson NR, Strugnell RA (2017). Gastrointestinal carriage is a major reservoir of Klebsiella pneumoniae infection in intensive care patients. Clin Infect Dis.

[65] Sakellariou C, Gürntke S, Steinmetz I, Kohler C, Pfeifer Y, Gastmeier P (2016). Sepsis caused by extended-spectrum beta-lactamase (ESBL)-Positive K. pneumoniae and E. coli: comparison of severity of sepsis, delay of anti-infective therapy and ESBL genotype. Yam WC, editor. PLoS One.

[66] Falcone M, Vena A, Mezzatesta ML, Gona F, Caio C, Goldoni P (2014). Role of empirical and targeted therapy in hospitalized patients with bloodstream infections caused by ESBL-producing Enterobacteriaceae. Ann Ig Med Prev E Comunita.

[67] Joo E-J, Park DA, Lee NR, Moon S-Y, Choi J-K, Ko J-H (2017). Impact of appropriateness of empiric therapy on outcomes in community-onset bacteremia by extended-spectrum-β-lactamase producing Escherichia coli and Klebisella pneumoniae definitively treated with carbapenems. Eur J Clin Microbiol Infect Dis.

[68] Vogelaers D, De Bels D, Forêt F, Cran S, Gilbert E, Schoonheydt K (2010). Patterns of antimicrobial therapy in severe nosocomial infections: empiric choices, proportion of appropriate therapy, and adaptation rates--a multicentre, observational survey in critically ill patients. Int J Antimicrob Agents.

[69] Harris PNA, Tambyah PA, Lye DC, Mo Y, Lee TH, Yilmaz M (2018). Effect of piperacillin-Tazobactam vs Meropenem on 30-day mortality for patients with *E coli* or *Klebsiella pneumoniae* bloodstream infection and ceftriaxone resistance: a randomized clinical trial. JAMA.

[70] Prevel R, Berdaï D, Boyer A (2019). Antibiotics for ceftriaxone-resistant Gram-negative bacterial bloodstream infections. JAMA.

[71] Boucher A, Meybeck A, Patoz P, Valette M, Thellier D, Delannoy PY (2016). Alternatives to carbapenems in ventilator-associated pneumonia due to ESBL-producing Enterobacteriaceae. J Inf Secur.

[72] Torres A, Zhong N, Pachl J, Timsit J-F, Kollef M, Chen Z (2018). Ceftazidime-avibactam versus meropenem in nosocomial pneumonia, including ventilator-associated pneumonia (REPROVE): a randomised, double-blind, phase 3 non-inferiority trial. Lancet Infect Dis.

[73] Wagenlehner FM, Umeh O, Steenbergen J, Yuan G, Darouiche RO (2015). Ceftolozane-tazobactam compared with levofloxacin in the treatment of complicated urinary-tract infections, including pyelonephritis: a randomised, double-blind, phase 3 trial (ASPECT-cUTI). Lancet.

[74] Timsit J-F, Pilmis B, Zahar J-R (2017). How should we treat hospital-acquired and ventilator-associated pneumonia caused by extended-spectrum β-lactamase–producing Enterobacteriaceae?. Semin Respir Crit Care Med.

[75] Dautzenberg MJD, Bayjanov JR, Leverstein-van Hall MA, Muller AE, Gelinck LBS, Jansen CL (2018). Dynamics of colistin and tobramycin resistance among Enterobacter cloacae during prolonged use of selective decontamination of the digestive tract. Antimicrob Resist Infect Control.

[76] Halaby T, Al Naiemi N, Kluytmans J, van der Palen J, Vandenbroucke-Grauls CMJE (2013). Emergence of colistin resistance in Enterobacteriaceae after the introduction of selective digestive tract decontamination in an intensive care unit. Antimicrob Agents Chemother.

[77] Al Naiemi N, Heddema ER, Bart A, de Jonge E, Vandenbroucke-Grauls CM, Savelkoul PHM (2006). Emergence of multidrug-resistant Gram-negative bacteria during selective decontamination of the digestive tract on an intensive care unit. J Antimicrob Chemother.

[78] Huttner B, Haustein T, Uckay I, Renzi G, Stewardson A, Schaerrer D, et al. Decolonization of intestinal carriage of extended-spectrum -lactamase-producing Enterobacteriaceae with oral colistin and neomycin: a randomized, double-blind, placebo-controlled trial. J Antimicrob Chemother. 2013; Available from: https://academic.oup.com/jac/article-lookup/doi/10.1093/jac/dkt174. [cited 2018 Oct 31]. 10.1093/jac/dkt174 23719234

[79] Gosalbes MJ, Vázquez-Castellanos JF, Angebault C, Woerther P-L, Ruppé E, Ferrús ML (2016). Carriage of Enterobacteria producing extended-spectrum β-lactamases and composition of the gut microbiota in an Amerindian community. Antimicrob Agents Chemother.

[80] Araos R, Tai AK, Snyder GM, Blaser MJ, D’Agata EMC (2016). Predominance of Lactobacillus spp. among patients who do not acquire multidrug-resistant organisms. Clin Infect Dis.

[81] Bilinski J, Grzesiowski P, Sorensen N, Madry K, Muszynski J, Robak K (2017). Fecal microbiota transplantation in patients with blood disorders inhibits gut colonization with antibiotic-resistant bacteria: results of a prospective, single-center study. Clin Infect Dis.

[82] Singh R, van Nood E, Nieuwdorp M, van Dam B, ten Berge IJM, Geerlings SE (2014). Donor feces infusion for eradication of extended Spectrum beta-lactamase producing Escherichia coli in a patient with end stage renal disease. Clin Microbiol Infect.

[83] Singh R, de Groot PF, Geerlings SE, Hodiamont CJ, Belzer C, Berge IJMT (2018). Fecal microbiota transplantation against intestinal colonization by extended spectrum beta-lactamase producing Enterobacteriaceae: a proof of principle study. BMC Res Notes.

[84] Huttner BD, de Lastours V, Wassenberg M, Maharshak N, Mauris A, Galperine T, et al. A five-day course of oral antibiotics followed by faecal transplantation to eradicate carriage of multidrug-resistant Enterobacteriaceae: a randomized clinical trial. Clin Microbiol Infect. 2019. 10.1016/j.cmi.2018.12.009. 10.1016/j.cmi.2018.12.009 30616014

[85] Ruppé E, Martin-Loeches I, Rouzé A, Levast B, Ferry T, Timsit J-F (2018). What’s new in restoring the gut microbiota in ICU patients? Potential role of faecal microbiota transplantation. Clin Microbiol Infect.

[86] Caballero S, Kim S, Carter RA, Leiner IM, Sušac B, Miller L (2017). Cooperating commensals restore colonization resistance to vancomycin-resistant enterococcus faecium. Cell Host Microbe.

[87] Piewngam P, Quiñones M, Thirakittiwatthana W, Yungyuen T, Otto M, Kiratisin P. Composition of the intestinal microbiota in extended-spectrum β-lactamase-producing Enterobacteriaceae carriers and non-carriers in Thailand. Int J Antimicrob Agents. 2018; Available from: https://linkinghub.elsevier.com/retrieve/pii/S0924857918303686. [cited 2018 Dec 20]. 10.1016/j.ijantimicag.2018.12.006 PMC6700749 30578963

