# Supplementary figures and images for: Is systematic fecal carriage screening of extended-spectrum beta-lactamase-producing Enterobacteriaceae still useful in intensive care unit: a systematic review
Source: Crit Care. 2019 May 14;23:170. doi: 10.1186/s13054-019-2460-3 (PMC6518813; doi:10.1186/s13054-019-2460-3)

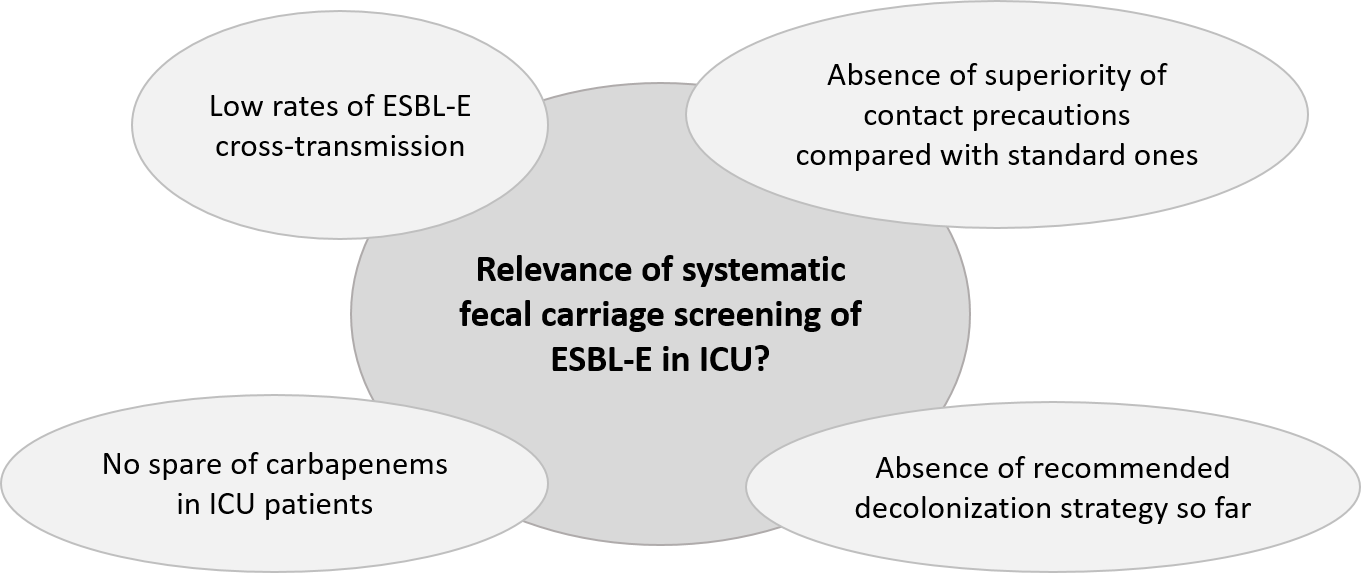

Supplement: Supplementary file 1 — Figure S1. Factors questioning the relevance of systematic fecal carriage screening of ESBL-E. ESBL-E: extended-spectrum beta-lactamase-producing Enterobacteriaceae. ICU: intensive care unit. (DOCX 98 kb) [file 13054_2019_2460_MOESM1_ESM.docx]
